# Characteristics of Antibiotic Resistance of Airborne *Staphylococcus* Isolated from Metro Stations

**DOI:** 10.3390/ijerph10062412

**Published:** 2013-06-13

**Authors:** Feng Zhou, Yuyan Wang

**Affiliations:** 1 Department of Clinical Medicine, School of Basic Medical Sciences, Fudan University, Shanghai 200032, China; E-Mail: 09301010100@fudan.edu.cn; 2 Department of Medical Microbiology and Parasitology, School of Basic Medical Sciences, Fudan University, Shanghai 200032, China

**Keywords:** *Staphylococci*, antibiotic susceptibility, *mecA*, *qac*, biofilm, metro stations

## Abstract

This study focused on the presence of antibiotic-resistant bacteria in a metro system as an example of a public transportation system. The molecular characteristics of *Staphylococcus* were investigated to discern which strains were isolated from metro stations in Shanghai. These were compared with strains isolated from hospital treatment rooms and parks. Airborne *Staphylococcus* samples in the metro were resistant to an average of 2.64 antibiotic types, and 58.0% of the strain samples were resistant to at least three antibiotics; this was a significantly higher rate than strains from the park, but was lower than those from hospitals. The presence of two antibiotic resistance genes of *Staphylococcus* strains, *mecA* (28.0%) and *qac* (40.0%), were also found at significantly higher levels in metro samples than park samples, but did not differ significantly from hospital samples. Furthermore, 22.0% of the metro *Staphylococcus* samples were found to be biofilm-positive. The high rate of antibiotic resistance found in *Staphylococcus* samples collected from metro stations, and the discovery of antibiotic-resistant genes, indicate that the closed indoor environment and crowded passengers may accelerate the spread of antibiotic resistant strains. More attention should be paid to the inspection and control of antibiotic resistant strains in public transportation systems.

## 1. Introduction

Antibiotic resistant strains are a global public health problem. Past reports have principally focused on the detection of antibiotic resistant bacteria in hospital acquired infections. Recently, with the discovery of multi-resistant strains in the broader community, public health officials have begun to realize the potential danger of the spread of these antibiotic resistant bacteria [1]. Public areas should be the subject of particular scrutiny, as antibiotic resistant strains are likely to spread through crowds and people in close contact in these areas. By now, antibiotic resistant strains can be found in public areas like schools [2], shopping centers or markets [3], offices [4] and so on. The inspection of antibiotic resistant strains in community was of important meaning to the control of community-acquired infections.

Transportation is often a major part of daily life, especially in large cities. With increasing demand for convenient and fast public transportation, the metro (also can be called subway and underground, a kind of railway transportation in cities.) is now principally used in major cities worldwide. By 2011, over 120 cities worldwide will have metro systems [5], but only a few cities have investigated the bacterial populations in the metro, including London [6], Cairo [7], Seoul [8], Tokyo [9], Beijing [10] and Oslo [5]. Antibiotic-resistant *Staphylococcus* was reported in the public transportation system in Portland (OR, USA) [11]. This suggested the possibility that antibiotic resistant strains can be spread through passenger flow in buses and trains. However, these studies have mainly focused on the amount of airborne bacteria, without an analysis of their molecular characteristics. Infectious diseases with high levels of pathogenicity are conspicuous targets for research, but normal flora with certain characteristics, such as antibiotic resistance, also require attention. Therefore, the main purpose of this current research was to detect antibiotic resistant strains in the Shanghai metro. 

As an average of 7 million passengers use the Shanghai metro daily, the metro stations have higher passenger volumes in an enclosed indoor environment compared with other public places such as schools and cinemas. Drug-resistant strains carried by passengers are likely to be spread through skin contact, respiration and body fluids in crowded carriages, with obvious public health implications. *Staphylococci* are the most common airborne strains in metro stations [5]. Coagulase-negative *Staphylococcus* (CNS) strains, such as *Staphylococcus epidermidis* and *Staphylococcus saprophyticus* are easily detected on the skin [12]. Direct contact, respiratory secretions and body fluids are all sources of the airborne bacteria found in metro stations. Growing numbers of reports have showed that CNS strains have a high rate of antibiotic resistance and research into the spread of antibiotic resistant CNS in the community is becoming increasingly important [13]. Besides, as these bacteria can develop biofilms and other specific adaptations [14], it can be more difficult for antibiotics to kill them effectively. Therefore, we investigated *Staphylococcus* strains isolated in metro stations in order to research the spread of antibiotic-resistant strains in public transportation systems.

In this study, the antimicrobial susceptibilities, antibiotic resistance genes and gene sequences of staphylococcal strains isolated from metro stations were investigated. Two genes for drug resistance were investigated, *mecA* and *qac*. The *mecA* gene encodes PBP-2α, which exists only in *Staphylococcus* strains [15], and has been identified in all methicillin-resistant CNS strains. Furthermore, this gene is easily transferred to all methicillin-resistant CNS, probably through transposons [16]. The *mecA* gene underlies resistance to lactam antibiotics and also confers resistance against other antibacterial drugs and metal ions [17]. Therefore, the *mecA* gene was detected as a marker to investigate antibiotic resistance in airborne CNS in metro stations.

The multi-drug-resistance gene, *qac*, is associated with the efflux of a number of classes of antimicrobial organic cations, including intercalating dyes, quaternary ammonium compounds, diamidines and biguanidines [18], which are components of most antiseptic compounds. The cleaning of metro stations is mainly disinfectant-based and, therefore, *qac* detection was used to investigate the efficiency of drug use in order to facilitate informed choices regarding the use of antibiotics and disinfectants. Finally, *Staphylococcus* is known to produce a biofilm which increases the likelihood of bacteria becoming antibiotic-resistant, so biofilm detection was included in this study too.

## 2. Materials and Methods

### 2.1. Sample Collection and Identification

Bacterial samples were collected from the waiting rooms of six different interchange metro stations, and compared with those of treatment rooms of six different hospitals and the grassland of two parks. Samples were then grown on nutrient agar plates using the settling process, as described by the Chinese Ministry of Public Health [19]. Briefly, the culture medium was nutrient agar (Microorganism Reagent Co. Ltd., Hangzhou, Zhejiang Province, China). After sterilization, the agar plates were placed 1.2 m above ground level and exposed for 5 min in the center of the waiting room. Nutrient agar plates were cultivated at 37 °C for 48 h.

All of the colonies on a quarter of the agar of each agar plate were identified to guarantee that the samples were selected at random. Based on the results of Gram-staining and microscopic examinations, biochemical investigations including catalase and plasma coagulase tests were conducted to determine the identity of the *Staphylococcus* strain. After the bacteria were purified by streak culture and re-confirmed by the above identification processes, the bacterial genome was prepared following DNA isolation by the TIANamp Bacteria DNA Kit (TianGen Co. Ltd., Beijing, China). The identity of the bacterial samples was confirmed by sequencing the 16S rRNA. The primers [20] were listed by following primers, forward: 5′-AGTCGAGCGAACAGATAAGGA-3′ and reverse: 5′-AAATGGTTACTCCACCGGCTT-3′, amplicon size, 1,403 bp. The amplification was performed using the following thermocycler parameters: initial denaturation at 95 °C for 3 min followed by 30 cycles of 30 s at 94 °C, annealing for 30 s at 54 °C and 60 s at 72 °C with a final extension for 10 min at 72 °C.

### 2.2. Antimicrobial Susceptibility Tests

Antimicrobial susceptibilities to 16 different antibiotics, including penicillin, oxacillin, gentamycin, netilmicin and 12 others (produced by Microorganism Reagent Co. Ltd., Hangzhou, Zhejiang Province, China), were determined by disk diffusion on nutrient agar plates according to the guidelines provided by the Clinical and Laboratory Standards Institute (CLSI). Briefly, each of the *Staphylococcus* samples was cultured overnight in liquid culture medium. The fresh bacterial culture medium was uniformly smeared on the agar plate. After that, disks with antibiotics were placed on the agar and cultivated at 37 °C for 18 h. The zone diameter breakpoints were measured to produce results, as judged by the CLSI guidelines.

### 2.3. Detection of Methicillin and Disinfectant Resistance Genes and Sequence Analysis

The *mecA* and *qac* genes were detected by PCR assays with the following primers: *mecA* gene, forward: 5′-GTTGTAGTTGTCGGGTTT-3′, reverse: 5′-CCACATTGTTTCGGTCTA-3′, amplicon size, 437 bp; *qac* gene, forward: 5′-CAGTTTGTAATTGGAGGC-3′; reverse: 5′-TTTCTTTGATAGCTGCTTG-3′, amplicon size, 429 bp. The amplification was performed using the following thermocycler parameters: initial denaturation at 94 °C for 3 min followed by 30 cycles of 30 s at 94 °C, annealing for 30 s at 55 °C and 30 s at 72 °C with a final extension for 5 min at 72 °C.

PCR products were separated by agarose gel (1.2%) electrophoresis at 55 V, stained with ethidium bromide, and de-stained in Tris-acetate-EDTA (TAE). Gels were photographed under UV transillumination. PCR products were sequenced by Jiesen Co. Ltd. (Shanghai, China) and sequences were analyzed with Vector NTI 10.0 and Mega 5.0 software. An unrooted phylogenetic tree was constructed using the neighbor-joining method and the Poisson correction model in Mega 5.05 [21]. The internal branching probabilities were determined by a bootstrap analysis with 1,000 replicates. Additionally, *mecA* and *qac* sequences from different sources were used to compare against the sample sequences. The dataset of the nucleotide sequences for staphylococcal strains were retrieved from the National Center for Biotechnology Information (NCBI) database. An alignment of all the sequences was performed using ClustalW 2.0 software [22]. 

### 2.4. Biofilm Detection

A semi-quantitative detection method was used to identify biofilm formation [23,24,25]. Briefly, fresh bacterial samples were mixed with new liquid nutrient medium in a 96-well plate (each strain sample was inoculated into four wells). The absorbance at 600 nm of the medium was adjusted to 0.01 to guarantee that all of the sample media had the same original concentration. After samples were cultivated for 24 h, they were washed in PBS four times. The samples were stained first with Bouin’s fixative for 1 h. After washing with phosphate-buffered saline, crystal violet was used as a second stain, and then samples in the wells of 96-well plates were washed gently with running water. Isolates were identified as biofilm-positive if the absorbance value at 570 nm differed significantly from the negative control strain (ATCC12228) (each strain sample was inoculated into four wells, thus, four absorbance values can be acquired. It would be compared with the control strains with *t*-test, *p* < 0.05). 

### 2.5. Statistical Analysis

The statistics were performed using SPSS 19.0 and GraphPad Prism 6 Demo software. SPSS 19.0 software was used to conduct statistical tests, including the Student’s *t*-test, chi-square test and Wilcoxon test. GraphPad Prism 6 Demo software was used to draw the figures and mark the statistical difference. 

## 3. Results

### 3.1. Strain Identification and Separation of CNS Strains

**Figure 1:**
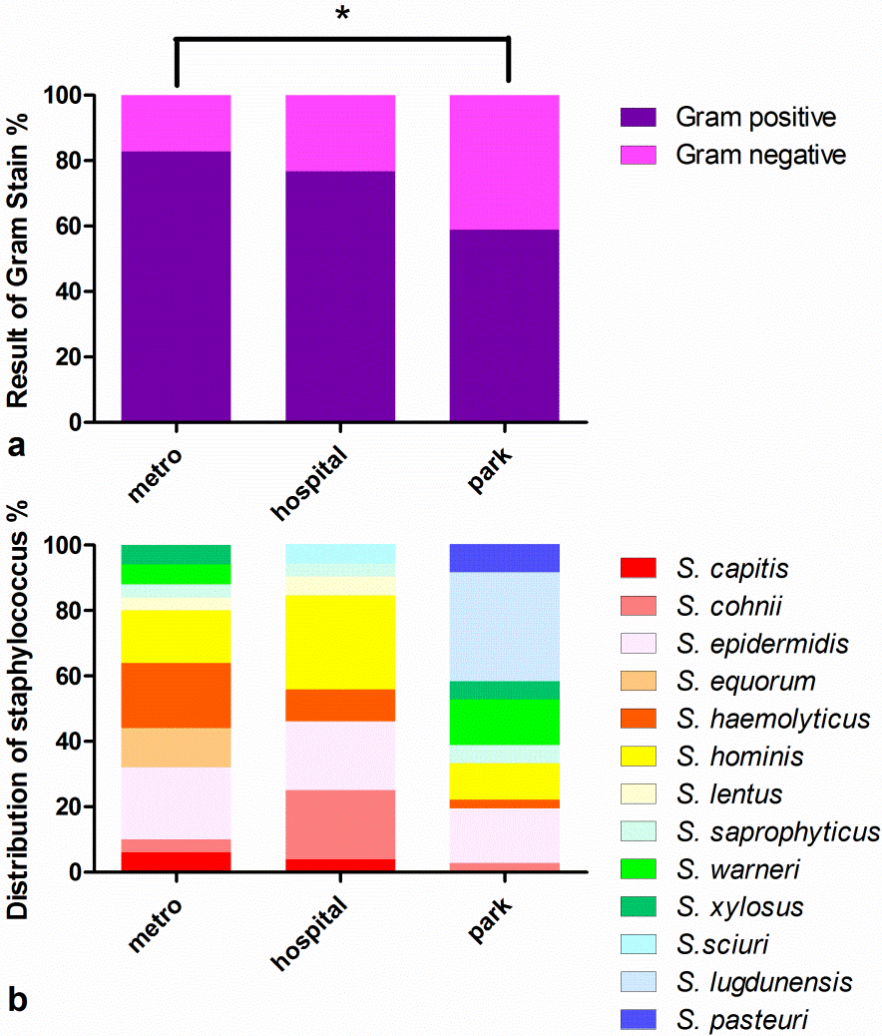
Distribution of strains. (**a**) The presence of Gram-positive straining in the park samples was significant lower (χ^2^ test, *p* < 0.05) than in the samples from the metro and hospital; (**b**) The distribution of *Staphylococcus* in the three groups. The composition and distribution of *Staphylococcus* strains differed between the three locations.

A total of 163 bacterial samples were obtained from metro stations, of which 135 (or 82.82%) were identified as Gram-positive bacteria. Overall, 69 out of 90 (76.67%) and 60 out of 102 (58.89%) bacterial colonies from hospital treatment rooms and parks, respectively, were Gram-positive. The prevalence of Gram-positive strains in parks was significant lower than in metro stations and treatment rooms (χ^2^ test, *p* < 0.05), but no significant difference was observed between metro stations and treatment rooms. Fifty staphylococcal samples from the metro station group (with 16S rRNA sequenced) were selected and 52 treatment room samples as well as 36 park samples were selected for further experiments. The distribution of specific *Staphylococcus* strains is shown in Figure 1(b).

### 3.2. Antimicrobial Susceptibility Tests

**Figure 2:**
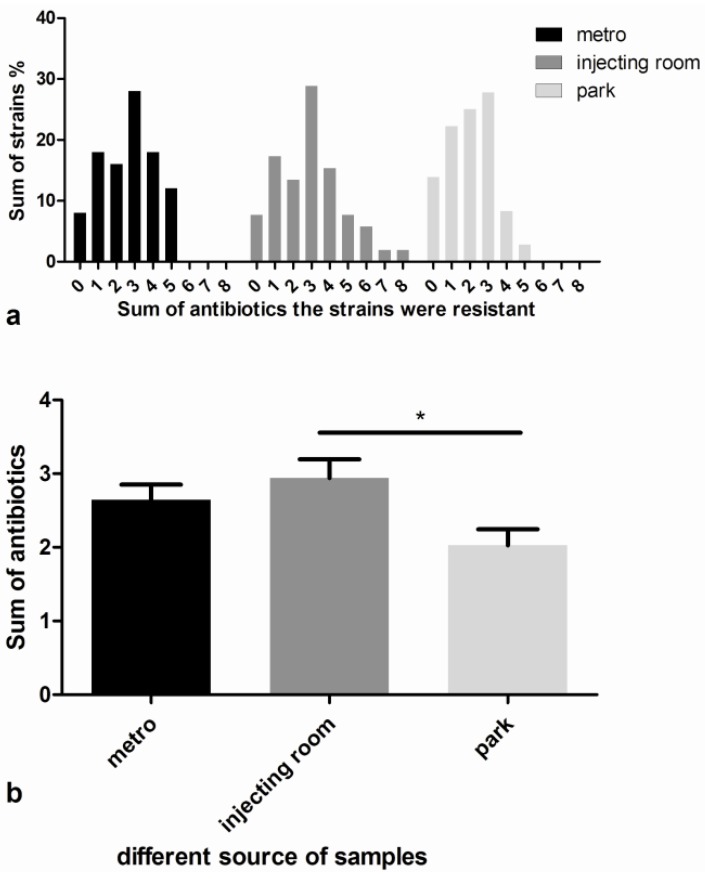
Statistical analysis of antibiotic-resistant strains. (**a**) The distribution of drug-resistant strains. The abscissa represents the number of antibiotics against which the CNS strains were resistant. The ordinates show the ratio of such strains in the total number of samples. The distribution of antibiotic-resistant strains showed a lower frequency of strains with resistance to more than three antibiotics in the park samples compared with those from the metro and hospital; (**b**) *Staphylococcus* samples from the park were resistant to an average of 2.03 antibiotic types, which was significantly lower (Student’s *t-*test, *p* < 0.05) than those from the metro (mean, 2.64) and hospital (mean, 2.94).

Antimicrobial susceptibility was evaluated by the disk diffusion assay and bacterial antibiotic resistance was analyzed according to the standards issued by the CLSI in 2010. The data showed a higher proportion of drug-resistant strains in treatment rooms and metro stations compared with those from parks. The highest frequencies of antibiotic resistance were detected against bactrim (38.95%), nitrofurantoin (36.28%) and penicillin (30.49%). Although most samples had resistance to some of the 16 antibiotics, strains isolated from different sources had different resistance to all antibiotics (Table 1). The distribution of the antibiotic resistant strains is shown in Figure 2(a). There was a higher frequency of strains with resistance to more than five antibiotics in treatment rooms compared with metro stations and parks. Over 90% of the *Staphylococcus* strains in metro stations and treatment rooms were resistant to at least one antibiotic. An oct-resistant strain from treatment rooms was identified. The distribution of antibiotic-resistant strains showed a higher frequency of strains with resistance to more than three antibiotics in treatment rooms compared with metro stations. However, more strains with resistance to less than three antibiotics were observed in samples from parks compared other groups. The Wilcoxon test showed no significant difference (*p* > 0.05) in the distribution of antibiotic-resistant strains between the hospital and metro samples. However, a significant difference (*p* < 0.05) was found between the park and metro samples as well as the park and hospital samples. Figure 2b showed that a significant difference (Student’s *t*-test, *p* < 0.05) was observed in the number of antibiotics that the strains were resistant to between hospital and park samples. 

**Table 1:** Resistance to 16 antibiotics among bacterial samples from different sources.

Antibiotics	Metro	Hospital	Park	*p* value	Average antibiotic resistance
	n = 50	n = 52	n = 36		
	Resistance rate	Resistance rate	Resistance rate		
*Cell wall synthesis*					
Oxacillin	22.00%	21.15%	36.11%	*p* > 0.05	33.33%
Penicillin (G)	28.00%	38.46%	25.00%	*p* > 0.05	32.61%
Ampicillin	20.00%	36.54%	13.89%	*p* < 0.05 *	24.64%
Cefepime	0.00%	1.92%	0.00%	*p* > 0.05	0.72%
Vancomycin	0.00%	0.00%	0.00%	*p* > 0.05	0.00%
Amoxicillin/clavulanic acid	8.00%	19.23%	0.00%	*p* > 0.05	10.14%
*Synthesis of protein*					
Gentamicin	0.00%	0.00%	2.78%	*p* > 0.05	0.72%
Netilmicin	0.00%	0.00%	0.00%	*p* > 0.05	0.00%
Erythromycin	30.00%	30.77%	11.11%	*p* < 0.05 *	25.36%
Tetracycline	6.00%	5.77%	2.78%	*p* > 0.05	5.07%
Clindamycin	24.00%	23.08%	19.44%	*p* > 0.05	22.46%
*Synthesis of DNA*					
Ciprofloxacin	2.00%	1.92%	8.33%	*p* > 0.05	3.62%
Pefloxacin	28.00%	9.62%	27.78%	*p* < 0.05 *	21.01%
*Synthesis of folacin*					
Bactrim	44.00%	42.31%	30.56%	*p* > 0.05	39.86%
*Synthesis of RNA*					
Rifampicin	0.00%	3.85%	2.78%	*p* > 0.05	2.17%
* Others*					
Nitrofurantoin	52.00%	34.62%	22.22%	*p* < 0.05 *	37.68%

### 3.3. Detection of Methicillin and Disinfectant Resistance Genes

Methicillin and disinfectant resistance genes were detected by PCR (Table 2) and the positive products were sequenced. The presence of both the *qac* and *mecA* genes in the park samples was significantly lower than samples from the metro stations and treatment rooms (χ^2^ test, *p* < 0.05). However, no significant difference was found between treatment rooms and metro stations. 

**Table 2:** Results from the detection of methicillin and disinfectant resistance genes. The presence of the *mecA* and *qac* genes were both significantly lower in the park samples compared to those from the metro and hospital (*p* < 0.05).

Gene/Group	Metro station		Treatment room		Park		*p* value
	n = 50		n = 52		n = 36		
*mecA*	14	28.00%	10	19.23%	2	5.56%	*p* < 0.05
*qac*	20	40.00%	18	34.62%	2	5.56%	*p* < 0.05

### 3.4. Sequence Analysis of the Resistance Genes

**Figure 3:**
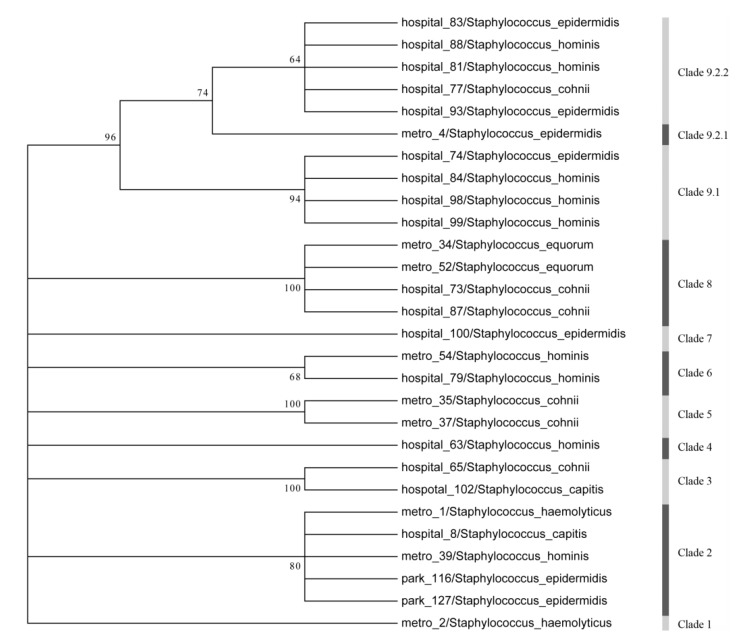
Phylogenetic analysis of *qac*. High levels of variability were found in *qac* gene fragment sequences from metro stations and treatment rooms. Samples from the same group were more similar.

The positive products of PCR were all sequenced. According to the BLAST result, the representative sequences were selected for further sequence analysis. The sequences were analyzed using Mega 5.0 and phylogenetic trees were constructed (Figure 3). 

The sequences of the *mecA* genes were shown to be highly conserved among the *Staphylococcus* strains identified in both metro stations and treatment rooms. BLAST analysis showed that *mecA* sequences had a higher rate of similarity and a lower aberration rate compared with reference sequences in GenBank, whereas the *qac* gene fragments were more variable. The phylogenetic tree showed that *qac* gene had nine phylogenetic clades (Figure 3). Furthermore, sequences from the hospital had a higher degree of similarity, although the hospitals were distributed throughout the city. Clades 3, 4, 7 and 9 mainly consisted of samples from the hospital, while samples from metro stations and parks were distributed separately. 

### 3.5. Biofilm Detection

**Figure 4:**
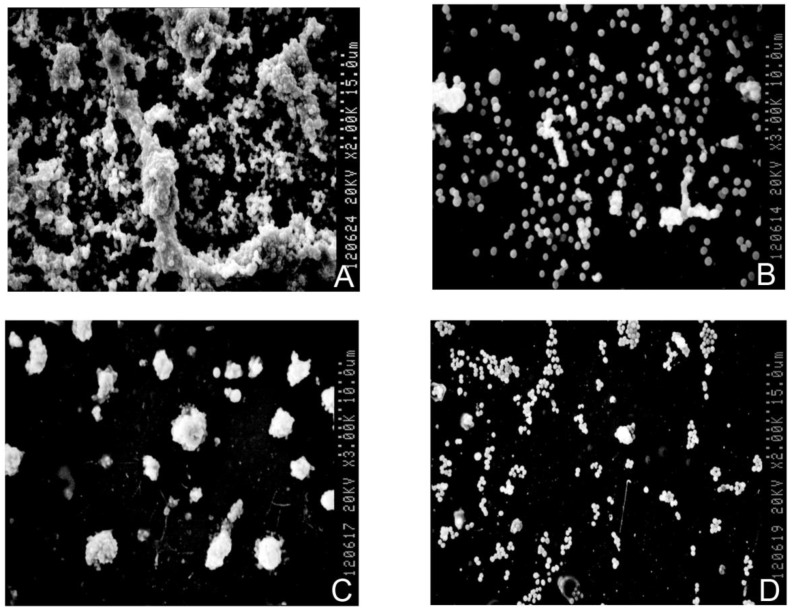
Electronic microscopy observations. The positive control strains A were shown to have agglomerated under scanning electronic microscopy. Similar results were found in the positive samples B. Both the negative control strains C and the negative samples D were separated from each other during scanning electronic microscopy.

The semi-quantitative detection showed that the frequency of biofilm-positivity in metro station bacterial strains was 22.00%, which was higher than in strains isolated from hospitals (15.38%) and parks (16.67%) but not significantly different (χ^2^ test, *p* > 0.05). The samples and the control strains had different shapes under scanning electron microscopy (Figure 4(a–d)). The positive control strains shown in Figure 4a became agglomerated, and the adhesions between the *Staphylococcus* strains were strong. The negative control strains shown in Figure 4(b) remained separated from each other. Figure 4(c,d) show two samples. Similar agglomerates to those in Figure 4(a) (positive control strain) are observed in Figure 4(c), which means that the positive sample had a similar shape to the positive control strain. Semi-quantitative detection also showed that the sample was biofilm-positive. Figure 4(d) shows that the negative sample strains were separated from each other and few agglomerates could be observed under scanning electron microscopy.

## 4. Discussion and Conclusions

With the appearance of antibiotic-resistant bacteria, increasing numbers of infections are causing huge losses to both economic concerns and social resources over recent decades, and this has become a global problem [26,27,28]. Although *Staphylococcus* strains, especially CNS, do not often cause disease, they can have strong levels of resistance to many antibiotics [29], and antibiotic resistance genes can accumulate in these strains. Thus, these bacteria have become an exchanger and have consequently spread antibiotic resistance genes to other strains. CNS strains, such as *Staphylococcus epidermidis* and *Staphylococcus saprophyticus*, are easily detected on human skin and mucous membranes as normal flora. The huge passenger flow in metro systems may provide ideal conditions for the widespread circulation of CNS strains.

Here, we used samples from a park and hospital as control groups to compare the level of antibiotic resistance of bacteria found in the metro. As an outdoor environment, the air circulates in parks significantly more than in the metro or hospital, and can therefore be regarded as a more natural environment. Both hospital and metro stations are indoor environments with crowds. No significant difference was found in either the level of antibiotic resistance or the distribution of antibiotic resistant strains between the hospital and metro samples, but both differed significantly when compared with the park samples. This indicated that higher levels of antibiotic resistance are found in closed crowded environments. Antibiotic resistance genes could be transferred between bacterial species by the exchange of genes and recombination events, such as transformation [30,31], transduction [32,33,34] and conjugation [35]. Thus, with increasing numbers of bacterial contacts, more antibiotic resistance genes can be acquired by a particular strain. The presence of the antibiotic resistance gene, *mecA*, and the anti-disinfectant gene, *qac*, were found at significantly higher levels in the metro and hospital samples than in those from the park, which may also have been a consequence of the crowded indoor environments. However, as there is a higher volume of people passing through the metro, the bacteria found here are more likely to have genes with high levels of mobility.

In our study, the antibiotics selected for sensitivity tests were all applicable to *Staphylococcus* and our results demonstrated that antibiotic resistance was present in the majority of bacteria obtained from metro stations. The samples showed different resistance profiles to different antibiotics. This was probably a result of the frequency of their use and the history of these different antibiotic types. Although most staphylococcal strains showed resistance to one or some drugs, the presence of multi-resistance was far lower than in those isolated from patients in other reports [36]. 

*Staphylococcus* strains with *mecA* are resistant to lactam antibiotics and frequently have multi-drug resistance, which may represent a serious health and economic concern [37,38]. Therefore, it is highly important to detect *mecA*, especially in *Staphylococcus* samples. In recent years, increasing numbers of reports have shown that the *mecA* gene is present in CNS strains, including hospital-acquired infections, neighborhoods [39,40], animal epidermis [41] and beaches [42], amongst others. To date, no reports have described the detection or discovery of *mecA* in airborne bacteria isolated from public transportation systems. Our research has shown that the presence of *mecA* from *Staphylococcus* was not as high as in strains from patients who have used antibiotics for long periods, which can reach over 90% in China [43]. Nevertheless, the metro is an important part of the Shanghai public transport system and should warrant closer inspection.

Quaternary ammonium compounds, diamidines and biguanidines are components of many antiseptic compounds such as benzalkonium bromide. These disinfectants are likely to be used for daily cleaning. The gene product of *qac* can help bacteria to resist these disinfectants, but this has not previously been assessed in strains isolated from public transportation system. The detection of *qac* has been reported in the food industry [44], animal husbandry [45] and hospital environments [46]. The efficiency of sterilization procedures employed in public areas should be improved by the informed selection of suitable disinfectants according to bacterial antibiotic resistance profiles.

Biofilms can be defined as communities of microorganisms that are attached to a surface [47]. The extracellular polymeric substance also acts as a physical barrier to the permeation and the action of antimicrobial agents [48]. *Staphylococcus* strains can form biofilms, and recent research has shown that the bacteria within biofilms are much more resistant to antibiotics than planktonic cells [49,50]. In this study, 22% of the samples from metro stations were positive for biofilm formation. Combined with the antimicrobial susceptibility test results, biofilms are likely to enhance bacterial antibiotic resistance. Furthermore, in the bacterial community, antibiotic resistance genes can be transferred more easily and spread across a wider area in biofilms. As biofilms can form more frequently in wet environments, metro stations in Shanghai provide an excellent opportunity for growth as air-conditioners function most of the time, and most stations are underground with inefficient ventilation facilities. Thus, it is important to detect the formation of *Staphylococcus* biofilms in metro stations. 

In conclusion, multi-resistant *Staphylococcus* and antibiotic resistant genes were found more commonly in metro stations than parks. The closed indoor environment and crowded passenger volumes may accelerate the spread of antibiotic resistant strains. More attention should be paid to the inspection and control of antibiotic resistant strains in public transportation systems.

## References

[1] Baba T., Takeuchi F., Kuroda M., Yuzawa H., Aoki K., Oguchi A., Nagai Y., Iwama N., Asano K., Naimi T. (2002). Genome and virulence determinants of high virulence community-acquired MRSA. Lancet.

[2] Sobhy N., Aly F., Abd El Kader O., Ghazal A., Elbaradei A. (2012). Community-acquired methicillin-resistant *Staphylococcus aureus* from skin and soft tissue infections (in a sample of Egyptian population): Analysis of *mec* gene and staphylococcal cassette chromosome. Braz. J. Infect. Dis..

[3] Nobile C., Costantino R., Bianco A., Pileggi C., Pavia M. (2013). Prevalence and pattern of antibiotic resistance of *Campylobacter* spp. in poultry meat in Southern Italy. Food Control.

[4] Reynolds K.A., Watt P.M., Boone S.A., Gerba C.P. (2005). Occurrence of bacteria and biochemical markers on public surfaces. Int. J. Environ. Health Res..

[5] Dybwad M., Granum P.E., Bruheim P., Blatny J.M. (2012). Characterization of airborne bacteria at an underground subway station. Appl. Environ. Microbiol..

[6] Gilleberg S.B., Faull J.L., Graeme-Cook K.A. (1998). A preliminary survey of aerial biocontaminants at six London underground stations. Int. Biodeterior. Biodegrad..

[7] Awad A.H.A. (2002). Environmental study in subway metro stations in Cairo, Egypt. J. Occup. Health.

[8] Hwang S.H., Yoon C.S., Ryu K.N., Paik S.Y., Cho J.H. (2010). Assessment of airborne environmental bacteria and related factors in 25 underground railway stations in Seoul, Korea. Atmos. Environ..

[9] Kawasaki T., Kyotani T., Ushiogi T., Izumi Y., Lee H., Hayakawa T. (2010). Distribution and identification of airborne fungi in railway stations in Tokyo, Japan. J. Occup. Health.

[10] Dong S., Yao M. (2010). Exposure assessment in Beijing, China: Biological agents, ultrafine particles, and lead. Environ. Monit. Assess..

[11] Yeh P., Simon D., Millar J., Alexander F., Franklin D. (2011). A diversity of antibiotic-resistant *Staphylococcus* spp. in a public transportation system. Osong Public Health Res. Perspect..

[12] Mandell G.L., Bennett J.E., Dolin R. (2000). Mandell, Dauglas and Bennett’s Principles and Practice of Infectious Diseases.

[13] Schreckenberger P.C., Ilendo E., Ristow K.L. (2004). Incidence of constitutive and inducible clindamycin resistance in *Staphylococcus aureus* and coagulase-negative staphylococci in a community and a tertiary care hospital. J. Clin. Microbiol..

[14] O’Toole G., Kaplan H.B., Kolter R. (2000). Biofilm formation as microbial development. Annu. Rev. Microbiol..

[15] Enright M.C., Robinson D.A., Randle G., Feil E.J., Grundmann H., Spratt B.G. (2002). The evolutionary history of methicillin-resistant *Staphylococcus aureus* (MRSA). Proc. Natl. Acad. Sci. USA.

[16] Koksal F., Yasar H., Samasti M. (2009). Antibiotic resistance patterns of coagulase-negative *Staphylococcus* strains isolated from blood cultures of septicemic patients in Turkey. Microbiol. Res..

[17] Poston S.M., Li Saw Hee F.L. (1991). Genetic characterisation of resistance to metal ions in methicillin-resistant *Staphylococcus aureus*: Elimination of resistance to cadmium, mercury and tetracycline with loss of methicillin resistance. J. Med. Microbiol..

[18] Mitchell B.A., Brown M.H., Skurray R.A. (1998). *QacA* Multidrug Efflux Pump from *Staphylococcus aureus*: Comparative analysis of resistance to diamidines, biguanidines, and guanylhydrazones. Antimicrob. Agents Chemother..

[19] Ministry of Health Law and Oversight Division (2002). Disinfection Technical Specifications.

[20] Onni T., Sanna G., Cubeddu G.P., Marogna G., Lollai S., Leori G., Tola S. (2010). Identification of coagulase-negative staphylococci isolated from ovine milk samples by PCR-RFLP of 16S rRNA and gap genes. Vet. Microbiol..

[21] Tamura K., Peterson D., Peterson N., Stecher G., Nei M., Kumar S. (2011). MEGA5: Molecular evolutionary genetics analysis using maximum likelihood, evolutionary distance, and maximum parsimony methods. Mol. Biol. Evol..

[22] Larkin M.A., Blackshields G., Brown N.P., Chenna R., McGettigan P.A., McWilliam H., Valentin F., Wallace I.M., Wilm A., Lopez R. (2007). ClustalW and ClustalX version 2. Bioinformatics.

[23] Li M., Wang X., Gao Q., Lu Y. (2009). Molecular characterization of *Staphylococcus epidermidis* strains isolated from a teaching hospital in Shanghai, China. J. Med. Microbiol..

[24] Wang L., Li M., Dong D., Bach T.H., Sturdevant D.E., Vuong C., Otto M., Gao Q. (2008). *SarZ* is a key regulator of biofilm formation and virulence in *Staphylococcus epidermidis*. J. Infect. Dis..

[25] Koskela A., Nilsdotter-Augustinsson Å., Persson L., Söderquist B. (2009). Prevalence of the *ica* operon and insertion sequence *IS256* among *Staphylococcus epidermidis* prosthetic joint infection isolates. Eur. J. Clin. Microbiol. Infect. Dis..

[26] (1996). The World Health Report.

[27] Cosgrove S.E., Sakoulas G., Perencevich E.N., Schwaber M.J., Karchmer A.W., Carmeli Y. (2003). Comparison of mortality associated with methicillin-resistant and methicillin-susceptible *Staphylococcus aureus* bacteremia: A meta-analysis. Clin. Infect. Dis..

[28] Harbarth S., Rutschmann O., Sudre P., Pittet D. (1998). Impact of methicillin resistance on the outcome of patients with bacteremia caused by *Staphylococcus aureus*. Arch. Intern. Med..

[29] Gillespie B.E., Headrick S.I., Boonyayatra S., Oliver S.P. (2009). Prevalence and persistence of coagulase-negative *Staphylococcus* species in three dairy research herds. Vet. Microbiol..

[30] Lorenz M.G., Wackernagel W. (1994). Bacterial gene transfer by natural genetic transformation in the environment. Microbiol. Rev..

[31] Zhu B. (2006). Degradation of plasmid and plant DNA in water microcosms monitored by natural transformation and real-time polymerase chain reaction (PCR). Water Res..

[32] Hyder S.L., Streitfeld M.M. (1978). Transfer of erythromycin resistance from clinically isolated lysogenic strains of *Streptococcus pyogenes* via their endogenous phage. J. Infect. Dis..

[33] Blahova J., Hupkova M., Babalova M., Krcmery V., Schafer V. (1993). Transduction of resistance to imipenem, aztreonam and ceftazidime in nosocomial strains of *Pseudomonas aeruginosa* by wild-type phages. Acta Virol..

[34] Banks D.J., Porcella S.F., Barbian K.D., Beres S.B., Philips L.E., Voyich J.M., DeLeo F.R., Martin J.M., Somerville G.A., Musser J.M. (2004). Progress toward characterization of the group A streptococcus metagenome: Complete genome sequence of a macrolide-resistant serotype M6 strain. J. Infect. Dis..

[35] Kuenne C., Voget S., Pischimarov J., Oehm S., Goesmann A., Daniel R., Hain T., Chakraborty T. (2010). Comparative analysis of plasmids in the genus *Listeria*. PLoS ONE.

[36] Luo M., Fei Y.E., Wang D.M., He J.L., Zhong H.Y. (2010). Analysis of coagulase-negative *Staphylococcus* from sputum. J. Modern Clin. Med..

[37] Cosgrove S.E., Sakoulas G., Perencevich E.N., Schwaber M.J., Karchmer A.W., Carmeli Y. (2003). Comparison of mortality associated with methicillin-resistant and methicillin-susceptible *Staphylococcus aureus* bacteremia: A meta-analysis. Clin. Infect. Dis..

[38] Harbarth S., Rutschmann O., Sudre P., Pittet D. (1998). Impact of methicillin resistance on the outcome of patients with bacteremia caused by *Staphylococcus aureus*. Arch. Intern. Med..

[39] Hisata K., Ito T., Matsunaga N., Komatsu M., Jin J., Li S., Watanabe S., Shimizu T., Hiramatsu K. (2011). Dissemination of multiple MRSA clones among community-associated methicillin-resistant *Staphylococcus aureus* infections from Japanese children with impetigo. J. Infect. Chemother..

[40] Barbier F., Ruppé E., Hernandez D., Lebeaux D., Francois P., Felix B., Desprez A., Maiga A., Woerther P.L., Gaillard K. (2010). Methicillin-resistant coagulase-negative staphylococci in the community: High homology of SCCmec IVa between *Staphylococcus epidermidis* and major clones of methicillin-resistant *Staphylococcus aureus*. J. Infect. Dis..

[41] Schnellmann C., Gerber V., Rossano A., Jaquier V., Panchaud Y., Doherr M.G., Thomann A., Straub R., Perreten V. (2006). Presence of new *MecA* and *mph*(C) variants conferring antibiotic resistance in *Staphylococcus* spp. isolated from the skin of horses before and after clinic admission. J. Clin. Microbiol..

[42] Soge O.O., Meschke J.S., No D.B., Roberts M.C. (2009). Characterization of methicillin-resistant *Staphylococcus aureus* and methicillin-resistant coagulase-negative *Staphylococcus* spp. Isolated from US West Coast public marine beaches. J. Antimicrob. Chemother..

[43] Wei W.P. (2010). Distribution and drug-resistance of 408 strains of coagulase negative *Staphylococcus*. China Prac. Med..

[44] Heir E., Sundheim G., Holck A.L. (1998). The *Staphylococcus qacH* gene product: A new member of the SMR family encoding multidrug resistance. FEMS Microbiol. Lett..

[45] Luthje P., von Kockritz-Blickwede M., Schwarz S. (2007). Identification and characterization of nine novel types of small staphylococcal plasmids carrying the lincosamide. J. Antimicrob. Chemother..

[46] Bjorland J., Steinum T., Sunde M., Waage S., Heir E. (2003). Novel plasmid-borne gene *qacJ* mediates resistance to quaternary ammonium compounds in equine *Staphylococcus aureus*, *Staphylococcus simulans*, and *Staphylococcus intermedius*. Antimicrob. Agents Chemother..

[47] O’Toole G., Kaplan H.B., Kolter R. (2000). Biofilm formation as microbial development. Annu. Rev. Microbiol..

[48] Siddiqui A.R., Bernstein J.M. (2010). Chronic wound infection: Facts and controversies. Clin. Dermatol..

[49] Donlan R.M., Costerton J.W. (2002). Biofilms: Survival mechanisms of clinically relevant microorganisms. Clin. Microbiol. Rev..

[50] Donlan R.M. (2001). Biofilm formation: A clinically relevant microbiological process. Clin. Infect. Dis..

