# *Correction*: Zhou, F.; Wang, Y. Characteristics of Antibiotic Resistance of Airborne *Staphylococcus* Isolated from Metro Stations. *Int. J. Environ. Res. Public Health* 2013, *10*, 2412-2426

**DOI:** 10.3390/ijerph10104667

**Published:** 2013-09-30

**Authors:** Feng Zhou, Yuyan Wang

**Affiliations:** 1 Department of Clinical Medicine, School of Basic Medical Sciences, Fudan University, Shanghai 200032, China; E-Mail: 09301010100@fudan.edu.cn; 2 Department of Medical Microbiology and Parasitology, School of Basic Medical Sciences, Fudan University, Shanghai 200032, China

The authors wish to add the following amendments and corrections to their paper published in IJERPH [1].

1. Page 2417, line 4–5, the sentence “*The highest frequencies of antibiotic resistance were detected against bactrim (38.95%), nitrofurantoin (36.28%) and penicillin (30.49%).*” should read ”*The highest frequencies of antibiotic resistance were detected against bactrim (39.86%), nitrofurantoin (37.68%) and penicillin (31.16%)*.”

2. Page 2417, Table 1, the first two values of the “average antibiotic resistance” column were incorrectly calculated. We have corrected this critical error. The updated Table 1 should be as follows:

**Table 1:** Resistance to 16 antibiotics among bacterial samples from different sources.

Antibiotics	Metro	Hospital	Park	*p* value	Average antibiotic resistance
	n = 50	n = 52	n = 36		
	Resistance rate	Resistance rate	Resistance rate		
*Cell wall synthesis*					
Oxacillin	22.00%	21.15%	36.11%	*p* > 0.05	25.36%
Penicillin (G)	28.00%	38.46%	25.00%	*p* > 0.05	31.16%
Ampicillin	20.00%	36.54%	13.89%	*p* < 0.05 *	24.64%
Cefepime	0.00%	1.92%	0.00%	*p* > 0.05	0.72%
Vancomycin	0.00%	0.00%	0.00%	*p* > 0.05	0.00%
Amoxicillin/clavulanic acid	8.00%	19.23%	0.00%	*p* > 0.05	10.14%

3. Page 2420, line 19, the order in the figure legend was wrong. The sentences “*The positive control strains A were shown to have agglomerated under scanning electronic microscopy. Similar results were found in the positive samples B. Both the negative control strains C and the negative samples D were separated from each other during scanning electronic microscopy.*” should read “*The positive control strains A were shown to have agglomerated under scanning electronic microscopy. Similar results were found in the positive samples C. Both the negative control strains B and the negative samples D were separated from each other during scanning electronic microscopy.*”

The authors apologize for the inconvenience.
